# Aptamer-targeting of Aleutian mink disease virus (AMDV) can be an effective strategy to inhibit virus replication

**DOI:** 10.1038/s41598-021-84223-8

**Published:** 2021-02-25

**Authors:** Taofeng Lu, Hui Zhang, Jie Zhou, Qin Ma, Wenzhuo Yan, Lili Zhao, Shuguang Wu, Hongyan Chen

**Affiliations:** 1 Institute for Laboratory Animal Research, Guizhou University of Traditional Chinese Medicine, Guiyang, 550025 China; 2 State Key Laboratory of Veterinary Biotechnology, Harbin Veterinary Research Institute, Chinese Academy of Agricultural Sciences, Harbin, 150069 China; 3 Shanghai Laboratory Animal Research Center, Shanghai, 201203 China

**Keywords:** DNA, Antivirals

## Abstract

Aleutian mink disease (AMD), which is caused by Aleutian mink disease virus (AMDV), is an important contagious disease for which no effective vaccine is yet available. AMD causes major economic losses for mink farmers globally and threatens some carnivores such as skunks, genets, foxes and raccoons. Aptamers have exciting potential for the diagnosis and/or treatment of infectious viral diseases, including AMD. Using a magnetic beads-based systemic evolution of ligands by exponential enrichment (SELEX) approach, we have developed aptamers with activity against AMDV after 10 rounds of selection. After incubation with the ADVa012 aptamer (4 μM) for 48 h, the concentration of AMDV in the supernatant of infected cells was 47% lower than in the supernatant of untreated cells, whereas a random library of aptamers has no effect. The half-life of ADVa012 was ~ 32 h, which is significantly longer than that of other aptamers. Sequences and three dimensions structural modeling of selected aptamers indicated that they fold into similar stem-loop structures, which may be a preferred structure for binding to the target protein. The ADVa012 aptamer was shown to have an effective and long-lasting inhibitory effect on viral production in vitro.

## Introduction

Aleutian mink disease (AMD), which is caused by Aleutian mink disease virus (AMDV), is an important contagious disease that causes major economic losses for mink farmers globally^1^. AMDV has been defined by the International Committee on Taxonomy of Viruses as a member of the genus *Amdoparvovirus*, within the family *Parvoviridae* and subfamily *Parvovirinae*^2,3^*.* The AMDV genome encodes two structural proteins (VP1 and VP2) and three nonstructural proteins (NS1, NS2 and NS3)^4,5^. The VP2 protein is the major immunogenic antigen and is involved in viral tropism, pathogenicity and host selection^6,7^.

AMDV is one of the major pathogens that restricts development of the mink breeding industry in China. Using a counter immunoelectrophoresis assay (CIEP), the seropositivity rate of AMDV in north-east China has been estimated to be 41.8%, with 7.6% of a seronegative population found to be PCR-positive^8–10^. So far, there is no fully effective vaccine against AMDV and only partial protection has been achieved^11–13^. AMDV is persistent in the environment, is considered to be resistant to various physical and chemical treatments^14^, and can be transmitted vertically from pregnant minks to offspring^15^, both by direct and indirect contact. The design and development of antiviral agents would thus be important in controlling AMD.

Aptamers are single-stranded DNA (ssDNA) or RNA oligonucleotides, which can specifically recognize and regulate the functions of their targets. Aptamers for various targets, including proteins, peptides^16^, bacteria^17^, viruses^18,19^, cells^20^ and small molecules^21^, have been selected in vitro using the systematic evolution of ligands by exponential enrichment strategy (SELEX). Aptamers have clinical value as diagnostic^22^ and therapeutic agents, are easy to prepare on a large-scale, can easily be modified with high batch fidelity, show pharmaceutical flexibility and are not immunogenic^23–25^. Specific aptamers can target viral proteins involved in different stages of the viral lifecycle, including adsorption, penetration, replication, maturation and even release of virions. Aptamers have exciting potential for the diagnosis and/or treatment of infectious viral diseases^26^. Some aptamers have already been reported to have potential applications as therapeutics for viral infections, e.g., human immunodeficiency virus-1^27,28^, hepatitis C virus^24,29^, influenza virus^30,31^, hepatitis B virus^18^, dengue virus 2^32^ and rabies virus^33^. We believe that aptamers can be used to supplement antibody treatments^34^. In this study, we obtained aptamers for AMDV VP2 protein using in vitro magnetic beads-based SELEX, and evaluated the antiviral activity of the selected aptamers in an AMDV infection assay. The identification of antiviral aptamers would represent an important development in the search for treatments for AMDV.

## Results

### Generation of aptamers against AMDV VP2 protein

**Figure 1:**
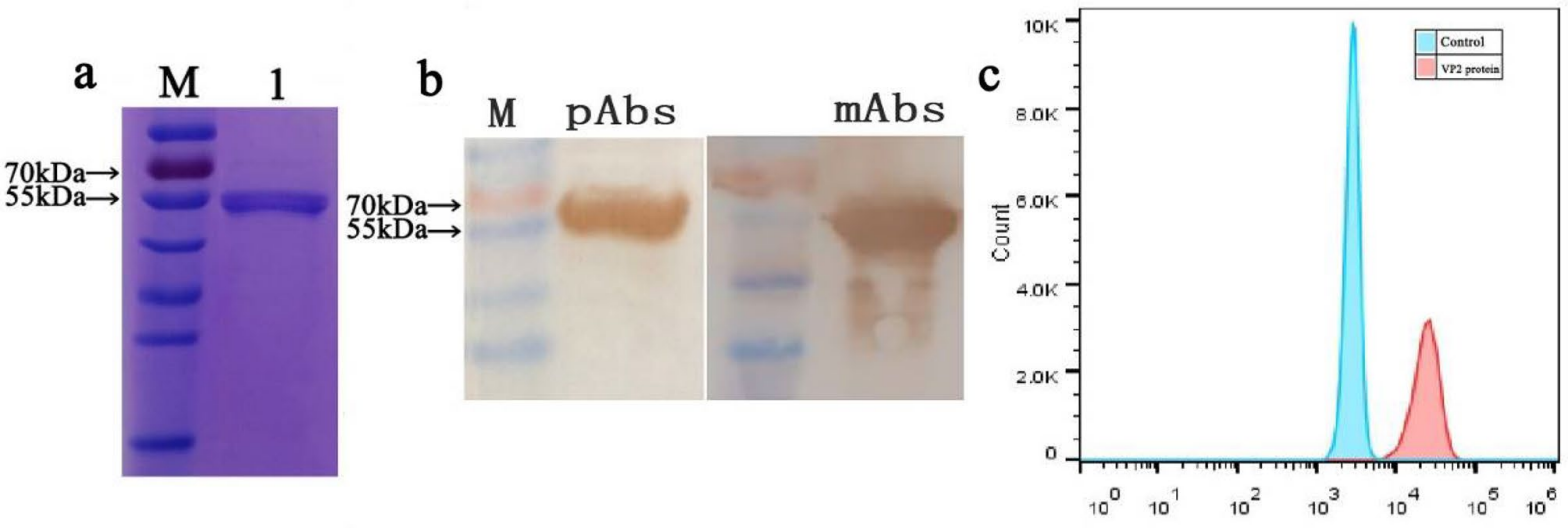
Preparation and quantification of AMDV VP2-modified magnetic beads. (**a**) Purified His-tagged AMDV VP2 protein detected by SDS-PAGE; (**b**) Purified VP2 protein recognized by VP2-specific mAbs and pAbs using western blot assay; (**c**) Flow cytometry showing efficiency of coupling of magnetic beads with VP2 protein.

Recombinant AMDV VP2 protein was expressed and purified by Ni-chelating affinity chromatography. The purified VP2 protein (aa: 200–588, ~ 55 kDa) was detected as a single band, with the expected molecular weight of ~ 55 kDa (Fig. 1a). The purified VP2 protein was also recognized by VP2-specific monoclonal antibodies (mAbs) and rabbit polyclonal antibody (pAbs) using a western blot assay (Fig. 1b). The full length gels and blots images was included in the Supplementary Information file.

The purified VP2 protein was coupled to Dynabeads MyOne Carboxylic Acid magnetic beads in sodium acetate buffer (pH 4.0). Using flow cytometry, the peak for magnetic beads coupled to VP2 protein was significantly right-shifted compared with that of blank magnetic beads (Fig. 1c). The magnetic beads coupled to recombinant AMDV VP2 protein can be used for target selection.

**Figure 2:**
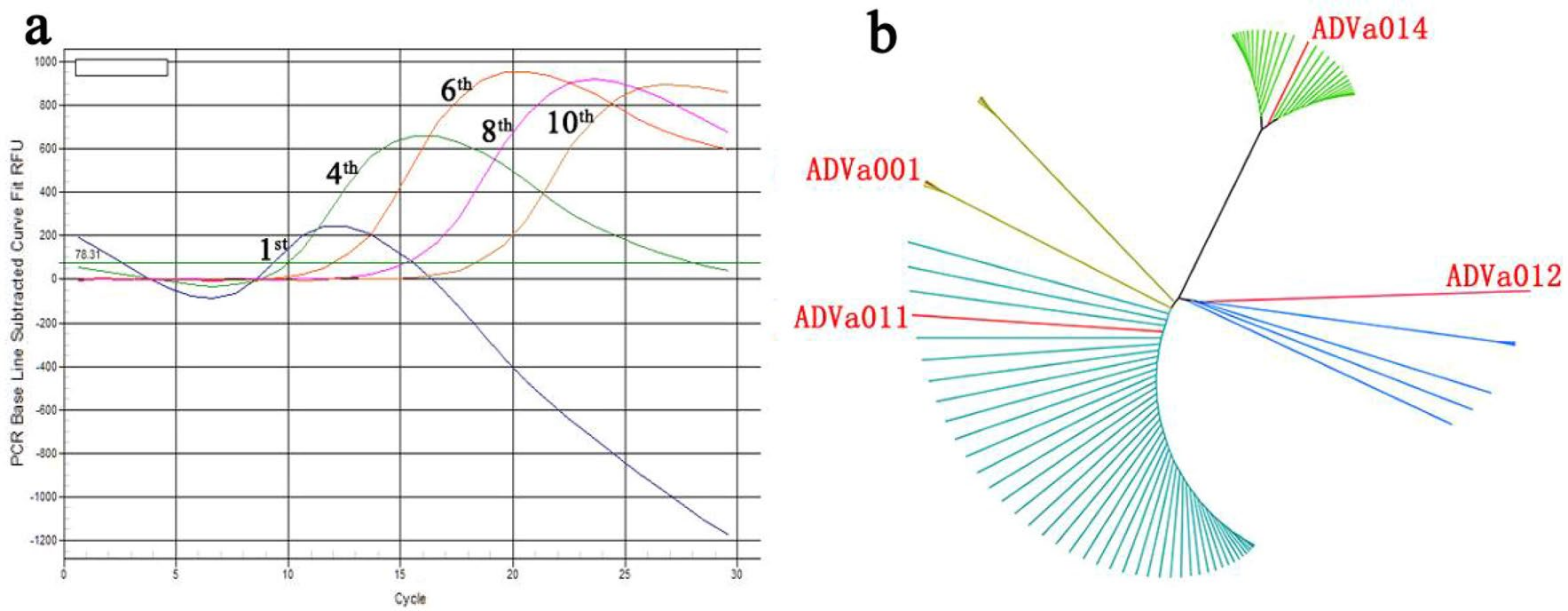
Generation of aptamers against AMDV VP2 protein. (**a**) Pools from the 1st, 4th, 6th, 8th and 10th selection rounds were used to monitor the SELEX process by quantitative real-time PCR; (**b**) Dendrogram analysis of the top 100 enriched aptamers after 10 rounds of SELEX, with the four selected aptamers shown as red lines. The phylogenetic tree was constructed using the program MEGA version 6.0 and was plotted using FigTree version 1.4.3 software (http://tree.bio.ed.ac.uk/).

SELEX was used to identify AMDV VP2-binding ssDNA aptamers from a random 40-nt ssDNA aptamer library consisting of ~ 10^14^ sequences. At every SELEX cycle, negative selection against canine parvovirus (CPV) VP2 protein-coupled magnetic beads was carried out to remove nonspecific bead-bound binders. The SELEX process leads to the enrichment of combined oligonucleotides and the diversity of the oligonucleotides is exponentially reduced during the process. The pools from the 1st, 4th, 6th, 8th and 10th selection cycles were used to monitor the SELEX process by quantitative real-time PCR (qPCR). We noticed that the plateau phase of the qPCR curves tended to be stable during the SELEX process (Fig. 2a). The sharply decreased amplification curves in the first few rounds indicate the high diversity of the pool, and the plateau phase amplification curves in the last few rounds indicate the reduced diversity of the pool. If the plateau phase of the qPCR curves from last few rounds tends to be stable, we can thus assume that the diversity of oligonucleotides was exponentially reducing during the SELEX process and that the screening process was successful. Our results are consistent with those of Luo et al.^35^.

After 10 rounds of selection, the aptamers from the 1st, 4th, 6th, 8th and 10th pool were sequenced by deep sequencing, using the Illumina HiSeq2000 platform, to identify specific aptamers for AMDV VP2. The top 100 enriched aptamers provided 40,725 sequences and sequences with > 1% frequency of occurrence are listed in Table 1. The enrichment reads number from the five pools was normalized and the enrichment tendency is also shown in Table 1. The enrichment data indicate that some aptamers, such as ADVa001, ADVa002, ADVa004, ADVa006, ADVa008, ADVa011, ADVa012 and ADVa014, were growing exponentially during the 10 selection rounds. The top 100 enriched aptamers were clustered using MEGA version 6.0 software, and the clustering tree was plotted using FigTree version 1.4.3 software (http://tree.bio.ed.ac.uk/) (Fig. 2b). Four major families were identified, and four aptamers (ADVa001, ADVa011, ADVa012 and ADVa014, drawn as red lines in Fig. 2b) from each clustering family were selected for further characterization.

**Table 1:** Aptamer sequences occurring with > 1% frequency in the top 100 enriched aptamers.

Aptamer	Sequences on random region (5′–3′)	The occurrence of aptamer (%)	The enrichment tendency
ADVa001	^a^AGCAGCACAGAGGTCAGATGGTGTGTGTGTGTGTGTGTGTGCCTATGCGTGCTACCGTGACCTATGCGTGCTACCGTGAA	10.04	
ADVa002	AGCAGCACAGAGGTCAGATGGTGTGTGTGTGTGTGTGTGCCTATGCGTGCTACCGTGAATCCTATGCGTGCTACCGTGAA	7.16	
ADVa003	AGCAGCACAGAGGTCAGATGGTGTGTGTGTGTGTGTGTGTGTGCCTATGCGTGCTACCGTCCTATGCGTGCTACCGTGAA	6.98	
ADVa004	AGCAGCACAGAGGTCAGATGGTGTGTGTGTGTGTGTGCCTATGCGTGCTACCGTGAATAGCCTATGCGTGCTACCGTGAA	3.46	
ADVa005	AGCAGCACAGAGGTCAGATGGTGTGTGTGTGTGTGTGCCTATGCGTGCTACCGTGAATTCCCTATGCGTGCTACCGTGAA	2.91	
ADVa010	AGCAGCACAGAGGTCAGATGACTAGCTGTCTCTGCCCTATGCGTGCTACCGTGAATTCACCCTATGCGTGCTACCGTGAA	2.18%	
ADVa007	AGCAGCACAGAGGTCAGATGGTGTGTGTGTGTGTGTGTGCCTATGCGTGCTACCGTGAAACCTATGCGTGCTACCGTGAA	1.99	
ADVa033	AGCAGCACAGAGGTCAGATGAGCTAGTGCCTATGCGTGCTACCGTGAATTCACGGTAGCACCTATGCGTGCTACCGTGAA	1.97	
ADVa009	AGCAGCACAGAGGTCAGATGGTGTGTGTGCCTTTGTGTGTGCCTATGCGTGCTACCGTGACCTATGCGTGCTACCGTGAA	1.86	
ADVa006	AGCAGCACAGAGGTCAGATGACTAGCTGTCTCTGCCCTATGCGTGCTACCGTGAATAGCGCCTATGCGTGCTACCGTGAA	1.80	
ADVa008	AGCAGCACAGAGGTCAGATGGTGTGTGTGTGTGTGCCTATGCGTGCTACCGTGAATAGCGCCTATGCGTGCTACCGTGAA	1.69	
ADVa011	AGCAGCACAGAGGTCAGATGGGGTTGTCGGTGTTTGCGGGCCTATGCGTGCTACCGTGAACCTATGCGTGCTACCGTGAA	1.65	
ADVa012	AGCAGCACAGAGGTCAGATGAGCTAGTGCCTATGCGTGCTACCGTGAATAGCGTCAGCAGCCTATGCGTGCTACCGTGAA	1.54	
ADVa013	AGCAGCACAGAGGTCAGATGGTGTGTGTGTGTGTGTGTGTGTGTGCCTATGCGTGCTACCCCTATGCGTGCTACCGTGAA	1.51	
ADVa015	AGCAGCACAGAGGTCAGATGGGTTGGTGGTGATTGTGTTGCCCTATGCGTGCTACCGTGACCTATGCGTGCTACCGTGAA	1.27	
ADVa016	AGCAGCACAGAGGTCAGATGGTGTGTGTGTGTGTGCCTATGCGTGCTACCGTGAATTCACCCTATGCGTGCTACCGTGAA	1.23	
ADVa019	AGCAGCACAGAGGTCAGATGGTGTGTGTGTGTGTGTGCCTATGCGTGCTACCGTGAAAGACCTATGCGTGCTACCGTGAA	1.20	
ADVa014	AGCAGCACAGAGGTCAGATGTGTTGTCCCGGGGCCCTATGCGTGCTACCGTGAATAGCGTCCTATGCGTGCTACCGTGAA	1.16	
ADVa018	AGCAGCACAGAGGTCAGATGGCCGGTCTCGTGGGACTGGGCCTATGCGTGCTACCGTGAACCTATGCGTGCTACCGTGAA	1.13	
ADVa017	AGCAGCACAGAGGTCAGATGGTGTGTGTGCCTGTGTGTGCCTATGCGTGCTACCGTGAATCCTATGCGTGCTACCGTGAA	1.09	
ADVa020	AGCAGCACAGAGGTCAGATGTGTTGTGCGGTTTGAGGGTGCCCTATGCGTGCTACCGTGACCTATGCGTGCTACCGTGAA	1.05	
ADVa021	AGCAGCACAGAGGTCAGATGGGGTTGAGTTGGTCGTGTGGCCTATGCGTGCTACCGTGAACCTATGCGTGCTACCGTGAA	1.04	

^a^Underlined bases indicate the constant regions.

### Characterization of aptamers

**Figure 3:**
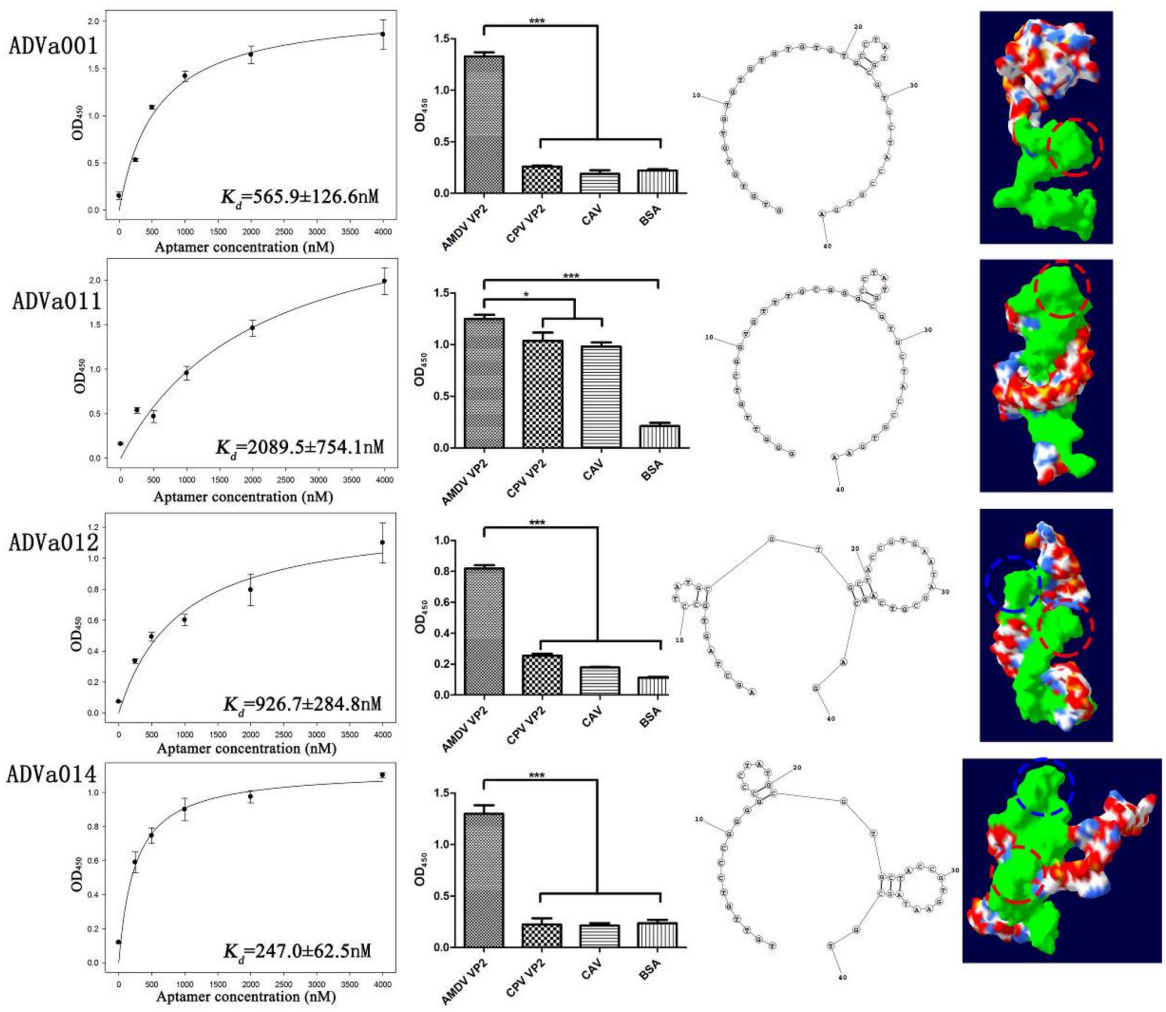
Characterization of aptamers. Calculated *K*_*d*_ values, measured by ELONA (565.9 ± 126.6 nM for ADVa001, 2089.5 ± 754.1 nM for ADVa011, 926.7 ± 284.8 nM for ADVa012 and 247.0 ± 62.5 nM for ADVa014), are shown in the first column. The resulting absorbance values (OD450) were normalized and plotted against the aptamer concentration using Sigma Plot version 12.0 software. Specificity of the four aptamers for AMDV VP2 protein and three counter targets, canine parvovirus VP2 protein, canine adenovirus (CAV) and Bull Serum Albumin (BSA) is shown in the second column. P values < 0.05 were considered to be statistically significant. Structural analyses of the four aptamers, performed using RNA structure 6.0 and 3dRNA-V2.0 online tools to predict the 2-D and 3-D structures, are shown in the last two columns. *P < 0.05; **P < 0.01. The secondary structures of the candidate aptamers were predicted using the RNAstructure online program (version 6.0.1) (http://rna.urmc.rochester.edu/). The 3D structures were predicted using the mfold (http://unafold.rna.albany.edu/) and 3dRNA-V2.0 (http://biophy.hust.edu.cn/home.html) website tools.

The *Kd* values of the four selected aptamers (ADVa001, ADVa011, ADVa012 and ADVa014) were measured using an enzyme-linked oligonucleotide assay (ELONA). The resulting mean values were normalized and plotted against the aptamer concentration using Sigma Blot software. The fitted *K*_*d*_ values of ADVa001, ADVa011, ADVa012 and ADVa014 were 565.9 ± 126.6 nM, 2089.5 ± 754.1 nM, 929.7 ± 284.8 nM and 247 ± 62.5 nM, respectively (Fig. 3).

To further demonstrate the specificity of the selected aptamers, ELONA was performed using the three counter targets, CPV VP2 protein, CAV and BSA. ADVa001, ADVa012 and ADVa014 were found to have good specificity (P < 0.01) for AMDV VP2 protein over CPV VP2 protein, CAV and BSA (Fig. 3), but ADVa011 had poor specificity. ADVa014 thus has good affinity and specificity, and can be used to distinguish between AMDV and CPV using ELONA.

The aptamer sequences, excluding the constant regions, were used to predict secondary structure. The sequences of ADVa001and ADVa011 show that the molecules have a 2-bp stem/4-base loop at the 5′ end. The secondary structure of ADVa012 shows a molecule with a 2-bp stem/4-base loop at the 5′ end and a 3-bp stem/15-base loop at the 3′ end, connected by two unpaired bases. The secondary structure of ADVa014 shows a molecule with a 2-bp stem/5-base loop at the 5′ end and a 3-bp stem/9-base loop at the 3′ end, also connected by two unpaired bases (Fig. 3).

We predicted the 3D structures of the four aptamers using the DNA model on the RNA structure 6.0 and 3dRNA-V2.0 web servers (http://biophy.hust.edu.cn/home.html). We found that all four aptamers can form a 5′ end stem-loop structure (red circles). ADVa012 and ADVa014 can additionally form a 3′ end stem-loop structure (blue circles) (Fig. 3). The two stem-loop motifs may be associated with the binding affinity of the aptamers.

### Inhibition of virus replication by ADVa012

**Figure 4:**
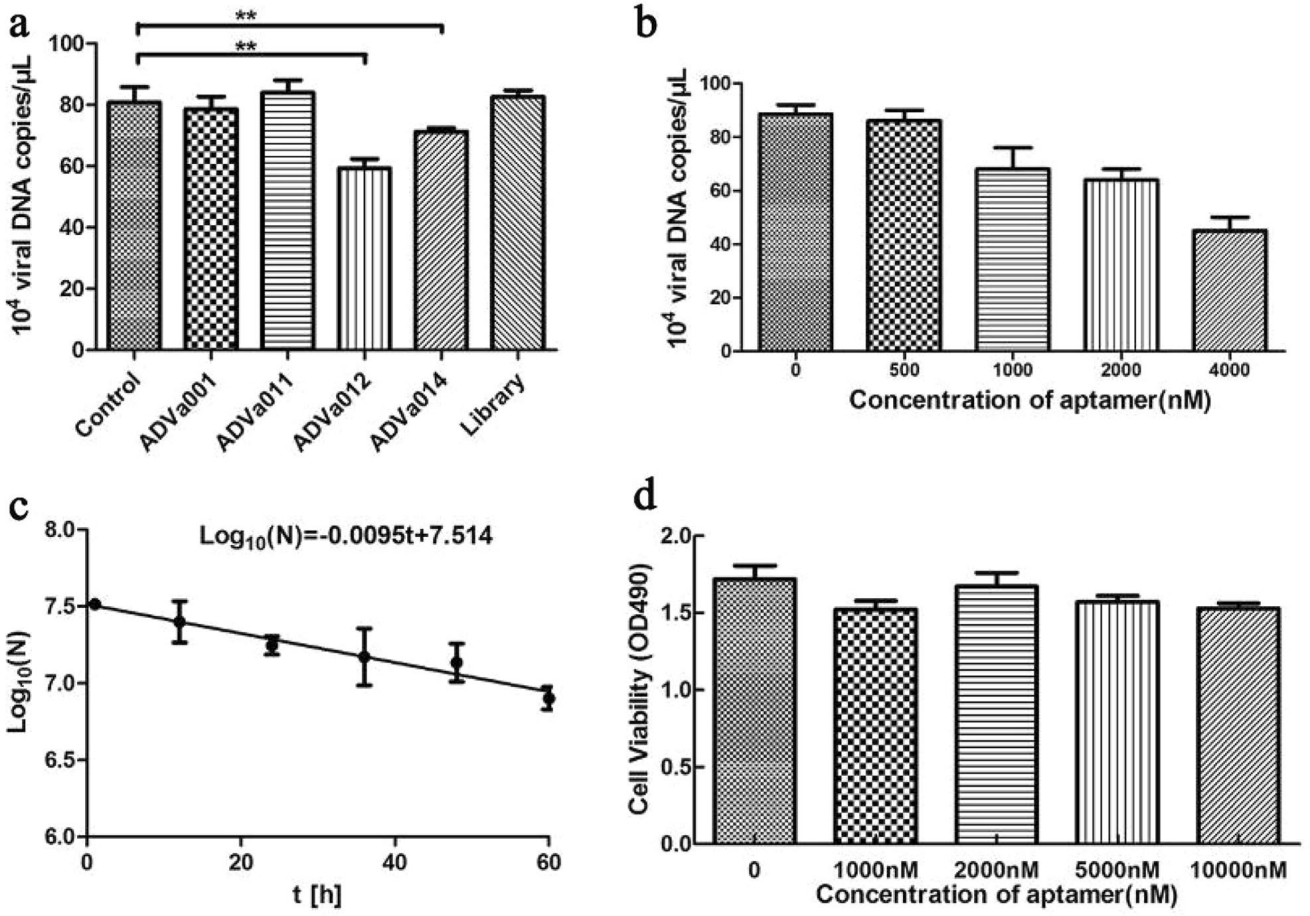
Inhibition of AMDV replication in CRFK cells by selected aptamers. (**a**) Inhibition of production of extracellular copies of viral DNA genome by selected aptamers. The infected cells were treated with the selected aptamer or library for 48 h, and copies number of viral DNA were measured by qPCR. The data represent the means of three different experiments; (**b**) Antiviral effect of different concentrations (0.5–4 μM) of ADVa012; (**c**) Determination of half-life of ADVa012 in CRFK cells. ADVa012 in the culture was quantified every 12 h by qPCR, and the experiment was performed three times; (**d**) Effects of ADVa012 on viability of CRFK cells. CRFK cells were treated with different concentrations (0–10 μM) of ADVa012 for 48 h and cell viability was determined using a CCK8 assay. The data were normalized with the control and represent means of three independent experiments. *P < 0.05; **P < 0.01.

After incubation for 1 h at 32 °C, AMDV-HLJ strain (MOI (Multiplicity of Infection) = 1,) and different aptamers (2 μM) were added simultaneously to Crandell-Rees Feline Kidney (CRFK) cells. At 48 h after addition of the ADVa012 aptamer, the amount of secreted virions corresponded to 5.9 × 10^5^ viral DNA genome copies/μL culture supernatant, as measured by qPCR. ADVa012 resulted in 32% lower virus concentration in the culture supernatant, compared with the blank control, whereas the virus concentration in the culture supernatant of cells treated with random library aptamers was have no significantly different to the blank control. ADVa014 showed weaker effects (12% inhibition) than ADVa012, ADVa001 and ADVa011 showed no significant inhibitory effect (Fig. 4a). ADVa012 dose-dependently reduced levels of AMDV over the concentration range 0.5–4 μM, and reduced levels of the virus by 47% at a concentration of 4 μM (Fig. 4b).

The half-life of ADVa012 in cell culture was determined by measuring the residual amount of the aptamer between 0 and 60 h after transfection. The aptamer was found to be exponentially degraded, with a half-life (t_1/2_) of log_10_(2)/0.0095 h≈32 h (Fig. 4c). A good inhibitory effect of ADVa012 on virus production can thus be expected in vitro. A CCK-8 assay showed no apparent cytotoxic effect (P > 0.05) in cells treated with different concentrations of ADVa012 (Fig. 4d).

### Identification of domain on VP2 protein recognized by aptamers

**Figure 5:**
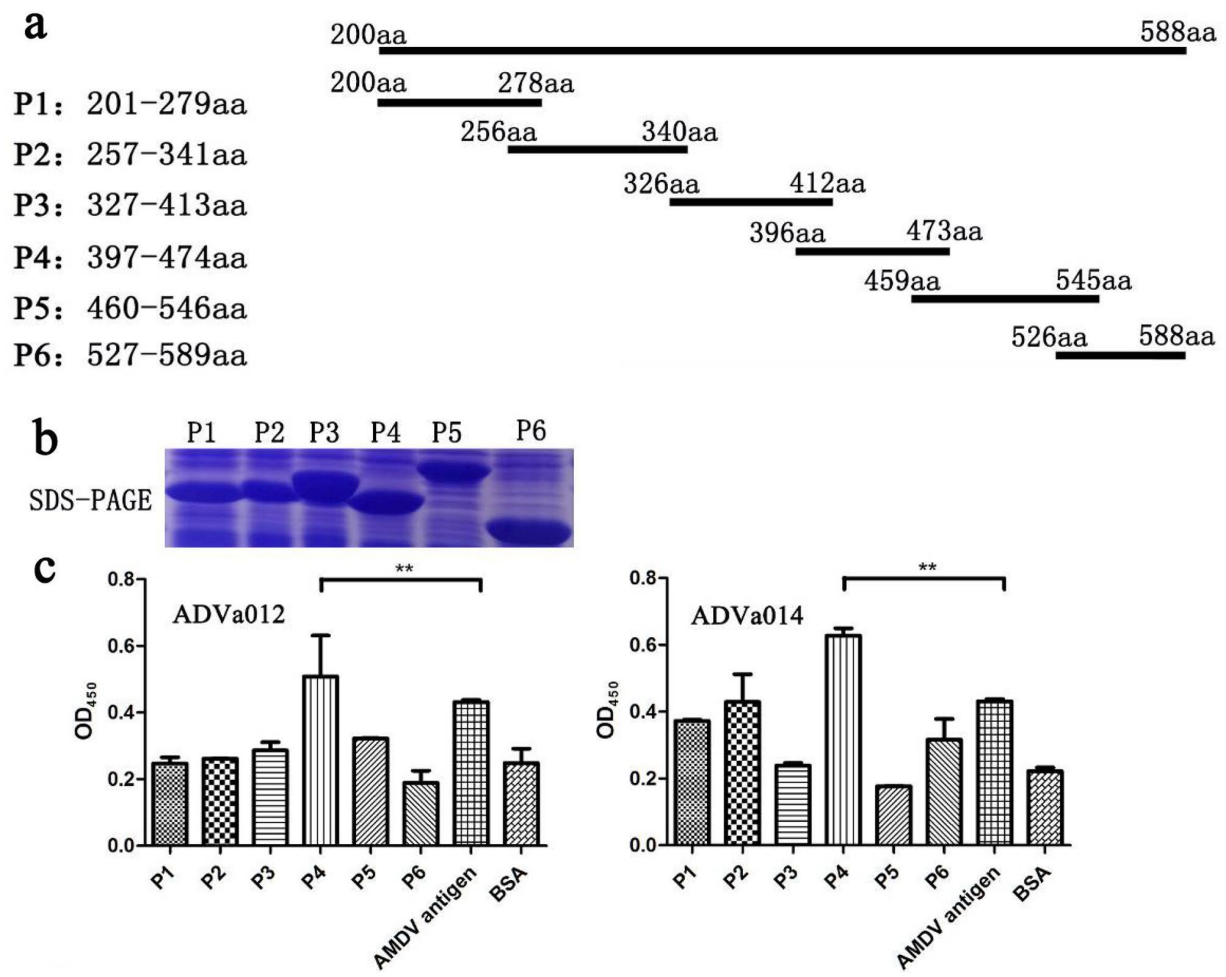
Domains on VP2 protein recognized by aptamers. (**a**) Six overlapping His-fused fragments spanning the VP2 protein were designed; (**b**) The six recombinant truncated His-fused proteins (P1 to P6) were expressed in a prokaryotic expression system; (**c**) Binding affinities of ADVa012 and ADVa014 for different domains of the VP2 protein were measured by ELONA. The six recombinant truncated His-fused proteins (P1 to P6) were coated onto the plates and biotin-labeled aptamers were added. The AMDV antigen and BSA were used as positive and negative controls. The P4 fragment is involved in binding to ADVa012 compared with AMDV antigen (P = 0.0025, P < 0.01), and is also involved in binding to ADVa014 in comparison with the AMDV antigen (P = 0.0041, P < 0.01). Results are the averages of three independent experiments performed in triplicate.*P < 0.05; **P < 0.01.

To identify the domain on the VP2 protein that is recognized by aptamers ADVa012 and ADVa014, six overlapping His-fused fragments spanning the VP2 protein were expressed and subjected to ELONA. Six recombinant truncated His-fused proteins were successfully expressed in a prokaryotic expression system (Fig. 5a,b). ELONA was used to determine the truncated fragments to which the aptamers bind.

The P4 fragment is involved in binding to ADVa012 (P < 0.01) and other fragments showed weaker binding, compared with AMDV antigen (P = 0.0025, P < 0.01, Fig. 5c). The P4 fragment is also involved in binding to ADVa014 in comparison with the AMDV antigen (P = 0.0041, P < 0.01), the P3, P5 and P6 fragments showed weaker binding effect (P < 0.01), and the P1 and P2 fragments showed the similar binding effect in comparison with the AMDV antigen (P > 0.05, Fig. 5c). These data suggest that the binding region for both ADVa012 and ADVa014 may be principally localized within the P4 fragment of the VP2 protein. We speculate, therefore, that the binding region recognized by ADVa012 and ADVa014 may be mainly located between aa residues 397 and 474 of the VP2 protein, and that residues within the P1 and P2 fragments may also contribute to binding to ADVa014.

## Discussion

Amdoparvoviruses cause severe diseases in a wide range of animals, resulting in major economic losses for farmers and threatening wild animal populations^2^. In adult mink, AMDV infects macrophages. Viral entry is probably mediated by cellular Fc receptors, which recognize antigen–antibody complexes and enhance viral entry into cells. This phenomenon, known as antibody-dependent enhancement^36^, means that immunosuppressed mink do not develop lesions when infected and is the reason for the failure of many vaccination strategies, although promising results have been obtained with an NS1 DNA vaccine^4^ and truncated mutant DNA vaccines^13^. There is, therefore, a pressing need to develop therapeutic agents for the prevention and control of AMD.

Efficient monitoring of the progress of SELEX is essential for successful selection of aptamers with good binding affinities^37^. We used qPCR to monitor the SELEX process and noticed that the diversity of oligonucleotides was exponentially reducing during the SELEX process. The sharply decreased qPCR curves indicate high diversity of the pool and the stable curves in the plateau phase indicate low diversity of the pool. We found that the plateau phase of the qPCR curves tended to be stable during the SELEX process and, if the plateau phase from the final ssDNA pool is stable, we can assume that the screening process has been successful. This result is consistent with that of Luo et al.^35^. We also noticed that PCR bias could be avoided by using the cycle threshold (Ct) values from the last pool sample to estimate the number of PCR cycles for the following round. In our experience, the use of C_t_ + 8 as the number of PCR cycles for the following round is suitable for SELEX experiments.

Although high-throughput sequencing (HTS) could not be performed in real-time, the purified DNA products from the 1st, 4th, 6th, 8th and 10th pools were analyzed by HTS. Bioinformatics analysis of the HTS data provided information about the number of sequences in the analyzed pools, their sequences, and enrichment of sequences during the SELEX process^38^. A comprehensive study of the data for five pools during the SELEX process was in agreement with the results obtained by qPCR. The HTS data on the enrichment tendency from the five pools indicated that many aptamers were growing exponentially during 10 rounds of selection, and some of these aptamers were shown to have high affinity and good specificity. The combination of HTS and bioinformatics analyses thus offered a more efficient tool for ensuring a high success rate in the selection of aptamers^39^.

The selected aptamers (ADVa012 and ADVa014) were found to specifically inhibit infection of CRFK cells by AMDV and to effectively reduce AMDV genome copy numbers in the infection system. At 48 h post-transfection, treatment with 1 μM ADVa012 led to a 32% lower concentration of virus in the culture supernatant and treatment with 4 μM ADVa012 led to a 47% lower concentration of virus. In contrast, after treatment with a random library of aptamers, the concentration of virus in the supernatant was not significantly different from that in the control group. ADV a012 dose-dependently reduced AMDV secretion in the infection system and had a half-life in CRFK cells of ~ 32 h in DMEM supplemented with 5% FBS at 37 °C under 5% CO_2._ In this study, we did not use full physiological conditions, just to improve the transfection efficiency using liposome transfection method, so we did not make direct comparison with other study. To validate our results, the experiments were performed three times, and the means of three duplicates were averaged and plotted. A strong and long lasting inhibitory effect of ADVa012 on virus production can thus be expected in vitro. It is also likely that ADVa012 could provide a treatment for AMDV. In this study, we just to explore an antiviral approach, without much consideration of practical application. After careful accounting, it costs about ¥300 to synthesize 10 nmol ADVa012-80 bp aptamer, so the cost of each treatment is between ¥20 and ¥30. That’s really high cost. But, if an industrial-grade synthetic process is used, the cost will reduce largely, and will be advantageous to extend and use the technology.

## Methods

### Reagents and viruses

The amine coupling kit was purchased from GE Healthcare (Chalfont St Giles, England), Carboxylic acid-dynabeads were purchased from Invitrogen (Carlsbad, CA, USA). SYPRO Orange protein gel stain was purchased from Life Technologies (Gaithersburg, MD, USA). The PCR components, including supermix and qPCR supermix, were purchased from TransGene Biological Science & Technology Company (Beijing, China). The UNIQ-10 oligonucleotide purification kit was purchased from Sangon Biotech (Beijing, China). Streptavidin-HRP was purchased from BD Biosciences (San Diego, CA, USA). Other reagents used in the study were purchased from Sigma-Aldrich (St. Louis, MO, USA). Solutions were prepared using ultrapure water (resistivity 18.2 Ω), purified using a Milli-Q system (Millipore, Madrid, Spain).

CRFK cells were grown in Dulbecco’s modified Eagle’s medium (HyClone, Logan, UT, USA), supplemented with 10% fetal bovine serum (FBS, HyClone) and 1% penicillin/streptomycin at 37 °C under 5% CO_2_. The AMDV-HLJ (GenBank accession# MF285607) strain was isolated, cultured and preserved in our laboratory^40^. The AMDV antigen (AMDV-G strain) was purchased from Jilin Teyan Biotechnology Co., Ltd. (Changchun, China). The CAVwas maintained in our laboratory. The recombinant AMDV VP2 and CPV VP2 proteins were expressed using the Escherichia coli expression vector pET30a (Qiagen, Hilden, Germany). Manipulation of microorganisms was carried out in a biosafety level II cabinet (BIO II A; Telstar Industrials, Terrassa, Spain).

### Expression and purification of recombinant AMDV VP2 protein

Recombinant AMDV VP2 protein was expressed using the *Escherichia coli* expression vector pET30a and the His-tagged protein was purified by Ni-chelating affinity chromatography, as described in detail in our previous study^40^. The production of purified recombinant VP2 protein was used to select aptamers in vitro. The mAbs and pAbs used in this study were maintained in our laboratory^41^.

### Oligonucleotide library and primers

An 80-base random ssDNA library (about 10^14^ oligonucleotides) containing 40 bases in random sequence flanked by defined primer-binding sites was synthesized by Comate Bioscience Co., Ltd. (Jilin, China) and purified by High Performance Liquid Chromatography (HPLC). The modified primers P1-F and P2- polyA-S, where F is 6-FAM, polyA is twenty poly (A) at the 5′ end and S is the spacer phosphoramidite 18, were synthesized by Comate Bioscience Co., Ltd. The forward primer P1-F and reverse primer P2-polyA-S were used to synthesize FAM-labeled double-stranded DNA (dsDNA) with a polyA tail in the antisense strand. The FAM-labeled dsDNA was heat-denatured and induced to produce ssDNA by 7 M urea-polyacrylamide gel electrophoresis (PAGE). The primers, P1 and P2, used to monitor the SELEX process by real-time-PCR, were synthesized by Comate Bioscience Co., Ltd. P1 and P2 were used with five forward primers with different barcodes (Lib3S1-01, Lib3S1-04, Lib3S1-06, Lib3S1-08 and Lib3S1-10) to produce the sequencing library. All random libraries and primers are listed in Table 2. The folding procedure for ssDNA was shown as below, 95 °C, 60 s; 95 °C, 90 s, − 1 °C per cycle, 70 cycles.

**Table 2:** Names and sequences of ssDNA library and oligonucleotide primers.

Name	Sequence information (5′–3′)
ssDNA library	AGCAGCACAGAGGTCAGATG-N40-CCTATGCGTGCTACCGTGAA
P1	AGCAGCACAGAGGTCAGATG
P2	TTCACGGTAGCACGCATAGG
P1-F	6FAM-AGCAGCACAGAGGTCAGATG
P2-polyA-S	A_20_-spacer18-TTCACGGTAGCACGCATAGG
Lib3S1-01	aaagcaAGCAGCACAGAGGTCAGATG
Lib3S1-04	aatgctAGCAGCACAGAGGTCAGATG
Lib3S1-06	accggcAGCAGCACAGAGGTCAGATG
Lib3S1-08	actggtAGCAGCACAGAGGTCAGATG
Lib3S1-10	agcgtcAGCAGCACAGAGGTCAGATG
AMDV-F	CCCTCCAGGTCAACTCTTTGTTAAA
AMDV-R	GCCTCTCCAAGTAAAGTAACCATAGG
ADVa012-80 bp	AGCAGCACAGAGGTCAGATGAGCTAGTGCCTATGCGTGCTACCGTGAATAGCGTCAGCAGCCTATGCGTGCTACCGTGAA

### Preparation and quantification of VP2-modified magnetic beads

Purified VP2 protein was coupled with Dynabeads MyOne Carboxylic Acid magnetic beads, using 1-ethyl-3-(3-dimethylaminopropyl)carbodiimide/*N*-hydroxysuccinimide. After washing with SELEX binding buffer (50 mM Tris, 100 mM NaCl, 5 mM KCl, 2 mM MgCl_2_, 1 mM CaCl_2_, 0.02% vol Tween 20, pH 7.4) three times, the coupled beads were suspended in SELEX binding buffer (200 uL) and stored at 4 °C.

To test the coupling efficiency, aliquots (2 μL) of protein-coupled beads were suspended in HEPES buffer (20 mM HEPES, 150 mM NaCl, 1 mM KCl, 1 mM CaCl_2_, 1 mM MgCl_2_, pH 7.4, 500 μL) containing 1 × Sypro orange protein gel stain. After heating for 5 min at 85 °C and supersonic treatment for 5 min, the extent of coupling was determined using an FC-500 flow cytometer (Beckman Coulter, Fullerton, CA, USA), with uncoupled blank magnetic beads as the negative control.

### SELEX protocol and process monitoring

At the beginning of the in vitro selection process, protein-coupled beads (50 μL, ~ 2.5 × 10^8^ beads) and ssDNA pool (500 pmoL) were incubated in SELEX binding buffer for 1 h at room temperature. After magnetic adsorption for 5 min and washing three times with binding buffer, the concentrated mixture was diluted to 100 μL using sterile both deionized and distilled water, and heated for 5 min at 100 °C. The collected elution mixture was used as a template for amplification of bound aptamers by PCR (15–25 cycles of 30 s at 95 °C, 30 s at 56 °C, 30 s at 72 °C, followed by 2 min at 72 °C) to obtain the dsDNA pool. After heat denaturation, the FAM-labeled ssDNA band was separated immediately from the 7 M urea-PAGE without nucleic acid dye. Following ethanol precipitation, the ssDNA was recycled and resuspended in sterile deionized distilled water. The selected ssDNA pool was used for the next round of selection. The target amount was reduced in successive cycles (2.5 × 10^8^ beads for the first three cycles, 1 × 10^8^ beads for the other cycles) to enhance the selection strength. The washing strength was also enhanced gradually by increasing the frequencies of washes (from three to five) to acquire aptamers with high affinity and specificity.

The SELEX process is the enrichment of combined oligonucleotides, and the diversity of oligonucleotides is an exponentially reducing process. Luo’s qPCR method^35^ was used to monitor the SELEX process. Ct values were measured for the samples and blank and used to evaluate amplification efficiency.

### Identification of aptamer sequences

After 10 rounds of selection, the ssDNA pool collected from the 1st, 4th, 6th, 8th and 10th SELEX rounds were amplified using different index-barcoded P1 primers (Lib3S1-01, Lib3S1-04, Lib3S1-06, Lib3S1-08 and Lib3S1-10) and P2 primer (Table 2). The final PCR products were purified using an UNIQ-10 oligonucleotide purification kit (Sangon Biotech, Shanghai, China). The purified products were pooled for quantification using a NanoDrop ND-100 spectrophotometer (Thermo Scientific, Waltham, MA, USA) and sequenced by the Deep Sequencing Group (Novegene, Beijing, China), using an Illumina HiSeq2000 platform (Illumina, Inc., San Diego, CA, USA) that generated 150 bp single reads.

The raw sequence data were then processed in FASTQ format for bioinformatics analysis using a Linux CentOS 6.3 cluster (4 cores, 64 GB). Among the obtained sequences, ssDNA aptamers with > 1% occurrence were clustered and subjected to further assessment. The top 100 enriched aptamers were clustered, and the phylogenetic tree was constructed using the maximum likelihood method in the program MEGA version 6.0. The clustering tree was plotted using FigTree version 1.4.3 software (http://tree.bio.ed.ac.uk/). The top enriched aptamers from each clustering family were selected as candidate aptamers for binding affinity and specificity studies.

The secondary structures of the candidate aptamers were predicted using the RNAstructure online program (version 6.0.1)^42^ (http://rna.urmc.rochester.edu/). To model the 3D structures of the candidate aptamers, the DNA sequences were transformed into RNA sequences, and the 3D structures were predicted using the mfold^43^ (http://unafold.rna.albany.edu/) and 3dRNA-V2.0^44,45^ (http://biophy.hust.edu.cn/home.html) website tools.

### Characterization of selected aptamers

The dissociation constants (Kd) of selected aptamers were measured by a modified ELONA^46,47^. After antigen coating and blocking steps, the biotinylated ssDNA aptamers were added at different concentrations (250–4000 nM) in binding buffer and incubated for 1 h at room temperature. HRP-conjugated streptavidin (1:2000; BD Company, San Diego, CA, USA) was then added and incubated for 30 min at 37 °C. After adding substrate and stop buffer, the optical density of 450 nm (OD_450_)was measured using a microplate reader (Bio-Tek Instruments, Inc., Winooski, VT, USA). The resulting absorbance values (OD_450_) were normalized and plotted against the aptamer concentration using Sigma Plot version 12.0 software. The *K*_*d*_ values of the different selected aptamers were estimated using the equation Y = B_max_*X*/(*K*_*d*_ + *X*). To determine the specificity of the selected aptamers, recombinant AMDV VP2, CPV VP2, CAV-1 and BSA were analyzed by ELONA. The mean of absorbance value (OD450) were calculated and analyzed by the non-parametric Student t-tests.

### Inhibition of AMDV production by selected aptamers

To investigate whether the aptamers could specifically inhibit AMDV production, the antiviral effect of four selected aptamers was measured. CRFK cells were infected with AMDV- HLJ strain, and viral genome copy numbers were used to represent viral titer. Briefly, AMDV-HLJ strain (MOI = 1) and different aptamers (2 μM) were added simultaneously to CRFK cells. The cells were incubated for 48 h at 32 °C after aptamer addition. The genomes of secreted virions were recovered by proteinase K digestion, followed by phenol–chloroform extraction. Viral genome copy numbers were measured using qPCR. The primers (AMDV-F and AMDV-R) are listed in Table 2. The antiviral effect of different concentrations (0.5–4 μM) of ADVa012 was measured to investigate dose-dependency. The ssDNA library was used as the negative control. The CRFK cell culture without aptamers or ssDNA library was used as blank control. Results from at least three independent assays for each aptamer were averaged. The mean of the viral genome copy numbers were calculated and analyzed by the one-way ANOVA with Tukey test for multiple comparisons.

### Cytotoxicity assays

The cytotoxicity of the aptamers was determined using a CCK-8 assay to measure cell viability. Before aptamer treatment, CRFK cells (1.0 × 10^5^ cells/well) were seeded in triplicate in a 96-well plates. The cells were then incubated with the aptamer (1–10 μM) for 48 h at 37 °C. Cell viability was determined using the CCK8 assay reagent (Beyotime Biotech, Beijing, China), according to the manufacturer’s instructions. After incubation for 3 h at 37 °C, the absorbance at 490 nm was measured using an ELISA plate reader. Three independent experiments were performed, and the result was calculated with respect to the control samples. The mean of absorbance value (OD_490_) were calculated and analyzed by the one-way ANOVA with Tukey test for multiple comparisons.

### Determination of half-life of ADVa012 in cell culture

The construct ADVa012-80 bp, consisting of the constant regions (Table 2), was used to determine the half-life of ADVa012 in cell culture. The aptamer was added to CRFK cells with 5% FBS at 37 °C in 24-well plates with 2 μg (5.9 × 10^13^ molecules) of ADVa012 per well using liposome transfection method. After 6 h, the cells were washed five times with pre-warmed PBS (500 μL) to remove non-internalized aptamers. The aptamers were harvested 1, 12, 24, 36, 48 and 60 h post-transfection by lysing the cells with lysis buffer (50 mM Tris–HCl, 100 mM NaCl, 20 mM EDTA, 0.5% *v/v* Nonidet P-40, pH 7.5, 200 μL per well). The aptamers were recovered by the phenol–chloroform method, and the recovered aptamers were quantified by qPCR, as described in the “SELEX protocol and process monitoring” section. The aptamer copy numbers were used to plot the half-life curve.

### Domain on VP2 protein recognized by aptamers

To identify the domain on the VP2 protein that is recognized by ADVa012 and ADVa014, six overlapping His-fused fragments spanning the VP2 protein were expressed and purified by Ni-chelating affinity chromatography. Information for the six recombinant truncated His-fused proteins is provided in Fig. 5a. ELONA was used to determine the truncated fragments to which the aptamers bind. Results are the averages of three independent experiments performed in triplicate. The mean of absorbance value (OD_450_) were calculated and analyzed by the one-way ANOVA with Tukey test for multiple comparisons.

### Statistics

Statistical analysis was carried out using GraphPad Prism version 5.01 (GraphPad Software, Inc. http://www.graphpad.com), and conducted by one-way ANOVA with Tukey test for multiple comparisons. P values < 0.05 were considered to be statistically significant.

## Supplementary Information

Supplementary Information.
