# Supplementary material for: Aptamer-targeting of Aleutian mink disease virus (AMDV) can be an effective strategy to inhibit virus replication
Source: Sci Rep. 2021 Feb 25;11:4649. doi: 10.1038/s41598-021-84223-8 (PMC7907208; doi:10.1038/s41598-021-84223-8)
Supplement: Supplementary file 1 — Supplementary Information. [file 41598_2021_84223_MOESM1_ESM.docx]

Aptamer-Targeted Aleutian mink disease virus (AMDV) Can Be an Effective Strategy to Inhibit Virus Reproduction

**Taofeng Lu^1#^, Hui Zhang^1#^, Jie Zhou^3^, Qin Ma^2^, Wenzhuo Yan^2^, Lili Zhao^2^, Shuguang Wu^1^, Hongyan Chen^2*^**

^1^ Institute for Laboratory Animal Research, Guizhou University of Traditional Chinese Medicine, Guiyang 550025, China

^2^ State Key Laboratory of Veterinary Biotechnology, Harbin Veterinary Research Institute, Chinese Academy of Agricultural Sciences, Harbin 150069, China

^3^ Shanghai Laboratory Animal Research Center, Shanghai 201203, China

^#^ These authors contributed equally to this work.

***^*^***corresponding. sydw2014@163.com


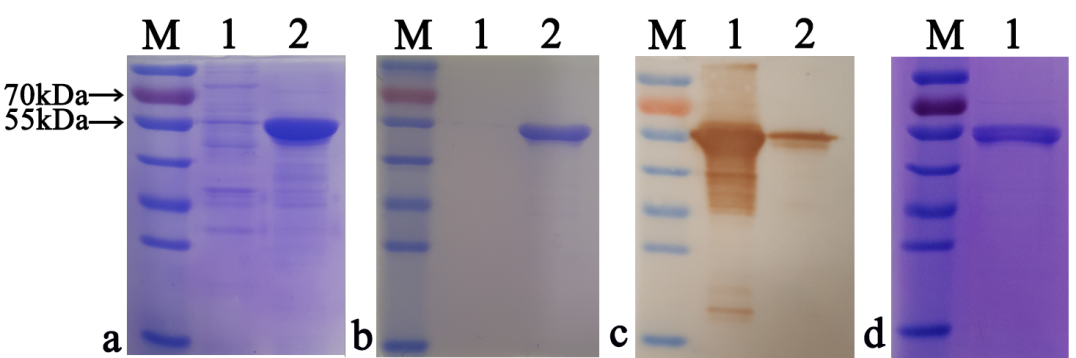


**Supplemental Figure 1**, Expression and purification of VP2 protein^41^. Bacterial lysates from E. coli BL-21 cells transformed with recombinant plasmid pET30a-VP2 were subjected to SDS-PAGE and western blot analysis. a VP2 protein expression with (Line1) or without (Line2) IPTG induction. b Expressed VP2 protein in the supernatant (Line1) and the inclusion body (Line2) of IPTG-induced E. coli cell lysate following ultrasonication. c WB analysis of VP2 protein in the supernatant (Line1) and the inclusion body (Line2) with anti-His mAb. d Purified recombinant protein VP2, Line 1. M: Standard molecular weight protein marker.


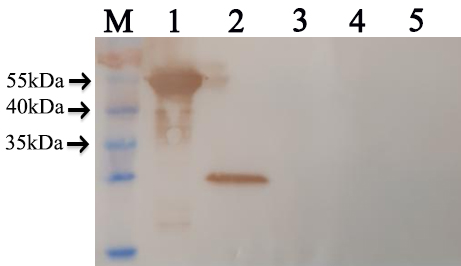


**Supplemental Figure 2**, Characterization of the monoclonal antibody. The antibody specificity was tested using WB assay: lane 1, recombinant VP2 protein; lane 2, the AMDV-G strain antigen; lane 3–5, MEV, CPV2 protein and the uninfected CRFK cell lysates; lane M, Standard molecular weight protein marker.


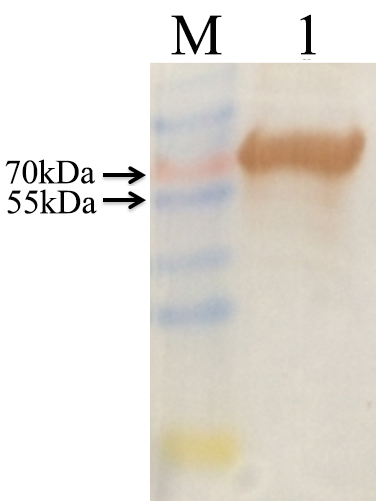


**Supplemental Figure 3**, Characterization of the rabbit polyclonal antibody. The antibody specificity was tested using WB assay: lane 1, purified recombinant VP2 protein; lane M, Standard molecular weight protein marker.


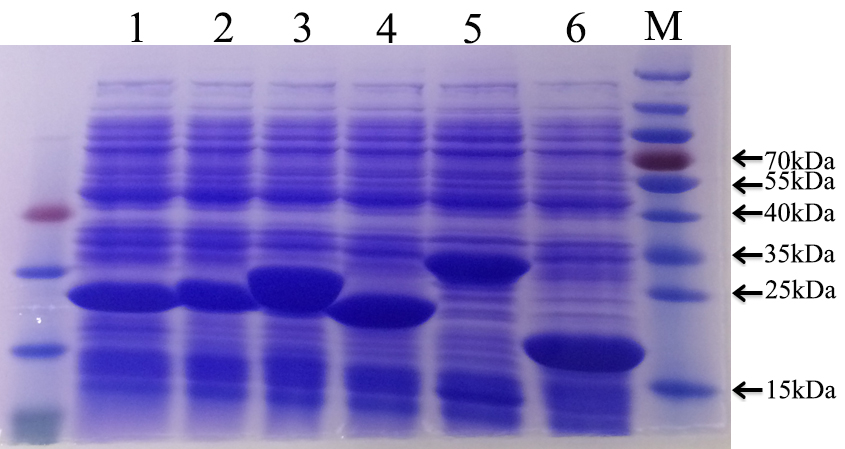


**Supplemental Figure 4**, The six recombinant truncated His-fused proteins (P1 to P6) were expressed in a prokaryotic expression system. lane 1, P1; lane 2, P2; lane 3, P3; lane 4, P4; lane 5, P5; lane 6, P6; lane M, Standard molecular weight protein marker.
